# Analytical Methodologies for Benzo[a]pyrene in Foods: A Review of Advances in Sample Preparation and Detection Techniques

**DOI:** 10.3390/foods15030591

**Published:** 2026-02-06

**Authors:** Di Yuan, Shan Zhang, Bin Hong, Shan Shan, Jingyi Zhang, Qi Wu, Dixin Sha, Shuwen Lu, Chuanying Ren

**Affiliations:** 1 Food Processing Research Institute, Heilongjiang Academy of Agricultural Sciences, Harbin 150086, China; yuandi199707@163.com (D.Y.); zhangshanfood@163.com (S.Z.); gru.hb@163.com (B.H.); 18845896856@163.com (S.S.); 18846080235@139.com (J.Z.); wuqi0322@163.com (Q.W.); shadixin1997@163.com (D.S.); 2 Heilongjiang Province Key Laboratory of Food Processing, Harbin 150086, China; 3 Heilongjiang Province Engineering Research Center of Whole Grain Nutritious Food, Harbin 150086, China

**Keywords:** benzo[a]pyrene, sample preparation, detection techniques, food safety

## Abstract

Benzo[a]pyrene (BaP), a potent carcinogenic polycyclic aromatic hydrocarbon, is a critical food contaminant originating from environmental deposition and thermal processing, posing a significant threat to public health and driving stringent global regulations. This review critically examines recent advancements in analytical methodologies for BaP determination, giving particular emphasis to sample preparation and detection techniques. The discussion covers the evolution from conventional methods, such as solid-phase extraction, towards more efficient and sustainable approaches, including magnetic, dispersive, and molecularly imprinted solid-phase extraction, as well as microextraction techniques and gel permeation chromatography. For detection, the performance of established chromatographic methods, such as gas chromatography–mass spectrometry (GC-MS) and high-performance liquid chromatography with fluorescence detection (HPLC-FLD), is evaluated against emerging rapid techniques such as sensors, immunoassays, and spectroscopic methods. The analysis reveals that while significant progress has been made in improving sensitivity, selectivity, and throughput, challenges remain in balancing speed with accuracy, managing matrix effects, and translating novel materials from research to routine application. The review concludes by underscoring the necessity for future development to focus on the integration of smart materials, automation, and advanced data science to achieve robust, on-site, and holistic monitoring solutions for ensuring food safety against BaP contamination.

## 1. Introduction

**Figure 1:**
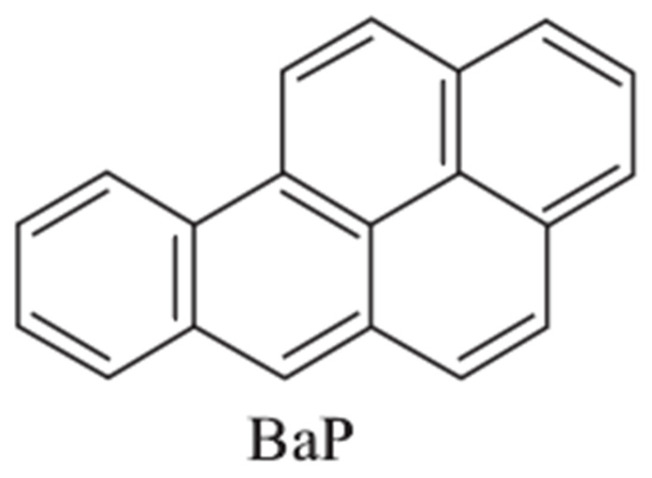
Molecular structure of benzo[a]pyrene.

Food safety constitutes a paramount global public health issue, wherein chemical contaminant residues emerge as a critical risk factor, provoking significant consumer health concerns and potentially leading to international trade disputes [1]. Among these contaminants, Polycyclic aromatic hydrocarbons (PAHs), a class of persistent organic pollutants widespread in the environment and food, have attracted considerable attention due to their well-documented carcinogenic, teratogenic, and mutagenic properties [2]. Among hundreds of PAH congeners, benzo[a]pyrene (BaP), a typical five-ring compound (Figure 1), is frequently employed as a characteristic marker and a primary monitoring target for assessing overall PAH pollution levels, owing to its exceptionally high carcinogenic potency [3]. This study’s focus is rooted in its historical role as a prototypical environmental carcinogen and its well-documented toxicological profile. From a regulatory and monitoring perspective, BaP is frequently used as a marker or indicator compound for the entire class of carcinogenic PAHs in food [4,5]. This is supported by the Toxic Equivalency Factor (TEF) approach, which estimates the overall carcinogenic potency of a PAH mixture relative to BaP (assigned a TEF of 1.0) [5]. Consequently, monitoring BaP provides a conservative and practical surrogate for risk assessment of PAH mixtures. BaP’s pronounced chemical stability and strong lipophilicity facilitate its accumulation along the food chain, particularly in lipid-rich food matrices. Contamination with BaP can be introduced at multiple stages of the food supply chain, spanning from agricultural production and environmental exposure to industrial processing (e.g., smoking, frying, drying, and roasting) and packaging/storage. Its formation mechanisms are complex, primarily involving the pyrolysis and pyrosynthesis of organic matter under high temperatures, making its control particularly challenging [6].

**Figure 2:**
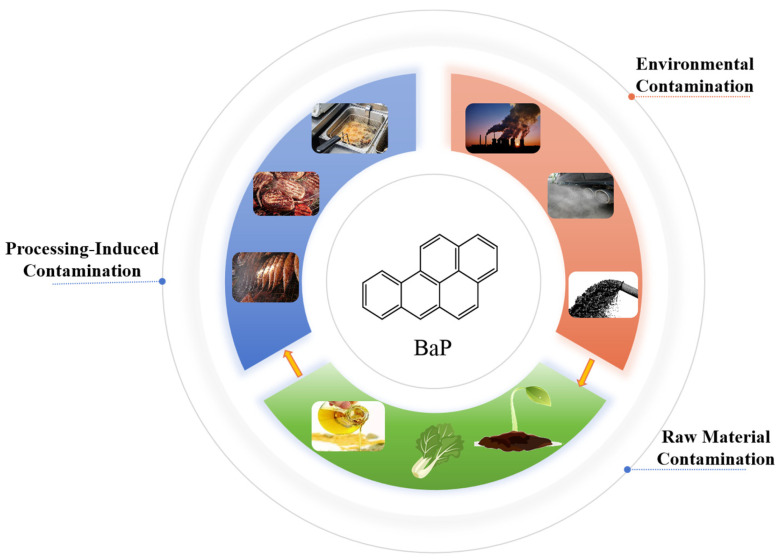
Main sources of benzo[a]pyrene in food.

The pervasive presence of BaP in food is a consequence of both environmental background contamination and processing-induced generation [7,8] (Figure 2). During raw material production, oil-bearing crops can accumulate BaP from diverse environmental sources, including industrial emissions, vehicle exhaust, and the combustion of tobacco (e.g., from cigarette butts littered in the environment) [9,10]. These contaminants reach agricultural fields via atmospheric deposition, soil adsorption, and irrigation with contaminated wastewater. The improper disposal of cigarette butts, which are known to leach BaP and other toxicants into soil and water, represents a notable point source of ecosystem contamination that can indirectly enter the food chain [11]. Recent research even suggests potential new risks from the application of biochar-based fertilizers [12]. More critically, thermal processing represents a major source of BaP formation. Complex chemical reactions, including fat pyrolysis, thermal polymerization, cyclization of Maillard reaction intermediates, and carbohydrate carbonization, contribute to its generation. Key factors such as processing temperature, duration, heat transfer mode (direct flame, hot air, oil medium), and fuel type significantly influence the final BaP levels, as notably observed in grilled and smoked meats, roasted coffee beans, and similar products [13,14,15]. For edible oils, the contamination level depends not only on the environmental BaP carried by the raw materials but also heavily depends on the processing techniques. Excessive pressing temperatures, poor quality extraction solvents, or insufficient refining and deodorization steps can all lead to elevated BaP residues, with crude oils that undergo minimal refining posing particularly high risks. Furthermore, storage, transportation, and packaging materials can contribute to secondary BaP introduction through migration or environmental cross-contamination. In the case of plastic packaging, BaP may originate as an inherent impurity from materials like carbon black or be formed during the high-temperature manufacturing of the polymers. It can then migrate into food, particularly into fatty products, upon direct contact [16,17]. Although the contribution from packaging and storage to the total BaP load is generally much lower compared to environmental and processing sources [18,19], it represents a continuous and direct route of contamination that is subject to increasing regulatory scrutiny for food contact materials.

Dietary intake serves as the primary route of human exposure to BaP. Extensive epidemiological studies in humans have established a strong link between dietary BaP intake and a significantly increased risk of various malignancies, including lung, stomach, bladder, and digestive tract cancers [20]. The severity of these health threats is underpinned by their toxicokinetics and dose–response relationship. The International Agency for Research on Cancer (IARC) has classified BaP as carcinogenic to humans (Group 1) based on sufficient evidence in humans and experimental animals [21]. Studies in experimental animals and in vitro systems have elucidated that the carcinogenic mechanism primarily hinges on metabolic activation: BaP is metabolized by cytochrome P450 enzymes (e.g., CYP1A1) into the highly reactive ultimate carcinogen, Benzo[a]pyrene-7,8-dihydrodiol-9,10-epoxide (BPDE) [22]. BPDE can form covalent adducts with DNA, leading to DNA damage, replication errors, and subsequently, gene mutations and cellular malignant transformation. Beyond this classical genotoxic pathway, recent animal studies have increasingly uncovered the role of epigenetic toxicity, including genome-wide DNA hypomethylation, hypermethylation of specific tumor suppressor gene promoters, and abnormal histone modifications. These epigenetic alterations regulate gene expression without changing the DNA sequence and play a crucial role in BaP-induced tumorigenesis [23]. Evidence from animal models and in vitro studies indicates that BaP can disrupt gut microbiota homeostasis, leading to reduced microbial diversity and functional dysbiosis, which may induce intestinal inflammation and systemic metabolic disorders, thereby increasing susceptibility to various diseases [24]. Research in animal models suggests cardiovascular toxicity, as BaP can synergize with a high-fat diet to promote vascular endothelial low-density lipoprotein accumulation and inhibit reverse cholesterol transport, accelerating atherosclerotic plaque formation. For example, in a key study, mice were administered BaP orally at doses of up to 12.5 mg per kilogram of body weight per day for 12 weeks to investigate this effect [25]. Animal studies demonstrate that BaP exhibits clear reproductive and developmental toxicity, adversely affecting spermatogenesis, oocyte maturation, and embryonic development. Studies employing relatively high doses, such as 50 mg per kilogram of body weight per day in rodent models, have been instrumental in elucidating the potency of such effects [26]. Furthermore, studies in animal models have shown that high doses of BaP demonstrate neurotoxicity, interfering with neurodevelopment and potentially causing cognitive and behavioral impairments [27]. Regarding the most sensitive toxicological endpoints, epidemiological studies most strongly associate long-term exposure with an increased risk of lung and skin cancers [21]. In contrast, oral exposure studies in animals, which form the basis of dietary risk assessments, identify tumors in the forestomach as a key sensitive endpoint [5]. This distinction is critical for risk assessment; while specific tumor sites may differ between species, the genotoxic mechanism is conserved. Consequently, quantitative risk assessments utilize dose–response data from these animal studies to inform human health protection levels, acknowledging that direct quantification of a dietary dose–response relationship in humans remains challenging due to complex, lifelong exposures [28]. In summary, BaP poses a multi-organ, systemic health threat, making it a significant food safety and public health concern.

Given this well-established risk profile, major regulatory bodies, including China, the European Union (EU), and the Codex Alimentarius Commission (CAC), have established stringent maximum limit (ML) standards for BaP in various food categories, reflecting a global trend towards increasingly stricter, more risk-based, and category-specific regulations [29,30,31]. A comparative overview is provided in Table 1. The EU generally enforces the most rigorous limits, exemplified by a 2.0 μg/kg ML for oils and fats, which is stricter than the corresponding CAC standard (5.0 μg/kg) and China’s current limit for oils (10.0 μg/kg), indicating an ongoing process of international harmonization. The scope of regulated food categories has expanded significantly from an initial focus on oils to now include cereals and their products, meat and meat products (especially smoked and grilled varieties), aquatic products, and food supplements. Differences in MLs primarily stem from variations in dietary patterns, environmental background levels, and outcomes of specific risk assessments, which are increasingly employed to scientifically underpin standard settings. These stringent regulatory standards, in turn, drive the advancement of analytical technologies, demanding lower detection limits, higher accuracy, and faster turnaround times. In China, the standard method for BaP determination is outlined in GB 5009.27-2016 [32], while the market regulatory authority has also issued a rapid detection method (KJ 201910 [33]) utilizing colloidal gold immunochromatography for on-site screening of BaP in edible oils, highlighting the need for diverse analytical solutions. Consequently, disparities in international BaP standards place higher demands on the global food trade, requiring exporters to vigilantly monitor regulatory changes in target markets and rely on robust analytical methods to ensure compliance.

Therefore, due to its singular toxicity, regulatory significance, and utility as an indicator, this review will focus specifically on the analytical methodologies for BaP itself, critically examining advances in its sample preparation and detection. The reliability of any detection technique is fundamentally contingent upon the effectiveness of the preliminary sample preparation, which aims to isolate the target analyte from interfering substances while minimizing losses. This review will therefore systematically critically evaluate the recent advances in this field in two core parts: first, by examining the evolution of extraction and purification techniques, which are crucial for obtaining a clean and concentrated analyte; and second, by discussing the principles and applications of the key detection methodologies that provide the necessary sensitivity and specificity for compliance and risk assessment.

## 2. Extraction and Purification Techniques

### 2.1. Solid-Phase Extraction (SPE)

SPE serves as a cornerstone technique for the purification and preconcentration of BaP from complex food matrices. The fundamental principle of SPE involves the selective retention of the target analyte onto a solid sorbent packed in a cartridge [34]. A typical operational sequence begins with conditioning the sorbent to activate its surface, followed by sample loading, where the analyte is adsorbed. A washing step is then applied to remove undesired matrix components, and finally, the purified analyte is eluted with a suitable solvent for subsequent analysis [35]. The continuous evolution of SPE has led to the development of various advanced formats, each offering distinct advantages for specific applications.

#### 2.1.1. Conventional Solid-Phase Extraction

Conventional SPE remains a mainstream purification method due to its strong selective adsorption capacity, ease of automation, good anti-interference ability, excellent reproducibility, and low detection limits. Its practicality is evidenced in various food matrices. For instance, Hu et al. [36] employed SPE as a key clean-up step to investigate the influence of frying conditions on the content of eight PAHs, including BaP, in sunflower seed oil. The method effectively isolated BaP from the complex oil matrix, revealing concentrations of 3.64–4.00 μg/kg, which exceeded the European Union’s maximum limit. In another application, Benny et al. [37] utilized an improved SPE method coupled with HPLC-FLD for the extraction and quantitative analysis of PAHs in plant samples like pecans and camellia. The reported high recovery rate for BaP (92–98%) underscores the method’s efficiency and accuracy in recovering the analyte from challenging botanical matrices. Despite its widespread use and advantages, conventional SPE faces notable limitations. A significant drawback is the limited universality of commonly used sorbents, often necessitating method-specific optimization for different food matrices. Furthermore, most commercial SPE columns are designed for single use, leading to substantial consumable costs in high-throughput laboratories. These practical and economic challenges, inherent to conventional SPE formats, have been recognized as key drivers for the development of more streamlined and cost-effective sample preparation approaches [38].

#### 2.1.2. Magnetic Solid-Phase Extraction (MSPE)

MSPE has emerged as an efficient and environmentally friendly sample preparation technique, leveraging functionalized magnetic nanoparticles (MNPs) as the sorbent. The extraction efficiency and selectivity of MSPE are predominantly governed by the surface chemistry of the engineered magnetic adsorbents. For non-polar compounds like BaP, the adsorption mechanisms primarily involve π-π interactions, hydrophobic effects, and van der Waals forces, making the strategic design of functional groups on the MNP surface critical [39]. A compelling application of this principle is the synthesis of a novel nanocomposite (Fe_3_O_4_@TCLN) for the determination of BaP in smoked foods, where Fe_3_O_4_ cores were modified with monomers of 4,4′-bipyridine and cyanuric chloride [40]. The aromatic rings in the 4,4′-bipyridine moiety were strategically utilized to provide abundant sites for π-π stacking interactions with the conjugated structure of BaP, thereby significantly enhancing the adsorption affinity and selectivity. When this nanocomposite was employed as an MSPE sorbent coupled with HPLC-FLD, the method demonstrated excellent performance, achieving a recovery of 94.74% for BaP, with remarkably low detection and quantification limits of 0.09 μg/kg and 0.28 μg/kg, respectively. The successful quantification of BaP in real smoked food samples, with an average content of 5.68 μg/kg, confirmed its practical utility for routine monitoring.

Despite these promising attributes, a critical consideration for MSPE is the potential vulnerability of the magnetic adsorbents to fouling or deactivation in exceptionally complex food matrices rich in proteins or fats, which could compromise long-term stability and reusability. Furthermore, while laboratory-scale synthesis of advanced MNPs shows great promise, the batch-to-batch reproducibility, scalability of production, and cost-effectiveness of these custom-designed materials need to be further addressed to facilitate their widespread adoption in routine analytical laboratories [41]. Therefore, despite being a highly efficient and green technique, the practical application of MSPE requires careful optimization and validation for each specific food matrix to ensure robust and reliable performance.

#### 2.1.3. Dispersive Solid-Phase Extraction (d-SPE)

Dispersive solid-phase extraction represents a streamlined and efficient approach for sample cleanup, characterized by the direct addition of a small amount of sorbent into the sample extract [42]. This mode maximizes the contact surface area between the sorbent and analytes through vigorous mixing (e.g., vortexing), facilitating rapid adsorption and subsequent removal of matrix interferences via centrifugation. Its simplicity and effectiveness have made it a cornerstone of modern, high-throughput methods like QuEChERS [43].

The performance of d-SPE is critically dependent on the selection and properties of the dispersive sorbent. Recent research has focused on developing novel materials with enhanced adsorption capacities. For instance, Lv et al. [44] employed a porous metal–organic framework, MIL-101(Cr), as a d-SPE sorbent for the purification of edible oils prior to BaP determination by HPLC-FLD. The high surface area and porosity of MIL-101(Cr) contributed to an efficient cleanup, achieving a low detection limit of 0.19 ng/mL and satisfactory recoveries of 88.8–118.8% across different oil matrices. This application highlights a key advantage of d-SPE based on advanced materials: a significant reduction in organic solvent consumption and a simplified workflow compared to conventional cartridge-based SPE. In another study, biochar derived from jujube seed shells, modified through KOH activation and acid treatment (K-H-BC), demonstrated a significantly enhanced adsorption capacity for BaP [45]. When applied to the d-SPE of tea beverages, this low-cost sorbent afforded a linear range of 25–500 µg/L and a limit of detection (LOD) at 2.12 µg/L, showcasing the potential of sustainable biochar materials in sample pretreatment.

Despite its operational advantages, the d-SPE technique faces challenges related to the universality and selectivity of sorbents. Commonly used primary sorbents like PSA and C18 may not sufficiently remove all classes of interferences in highly complex or unique food matrices, sometimes necessitating customized sorbent mixtures and optimization [46]. Furthermore, the manual centrifugation step, while simple, can become a bottleneck in ultra-high-throughput laboratories and may introduce variability. The future development of d-SPE is likely to focus on the rational design of more selective and robust sorbents, such as molecularly imprinted polymers or composite materials, to improve cleanup specificity [47]. Additionally, trends point towards the greater integration of d-SPE into automated or miniaturized analytical systems, further enhancing its efficiency and reproducibility for the routine monitoring of BaP and other contaminants.

#### 2.1.4. Molecularly Imprinted Solid-Phase Extraction (MISPE)

Molecularly imprinted solid-phase extraction represents a significant advancement in selective sample preparation, leveraging the unique properties of molecularly imprinted polymers (MIPs). These polymers are synthesized in the presence of a template molecule (the target analyte or a structural analog), creating tailor-made three-dimensional cavities that are complementary in size, shape, and chemical functionality. This process endows MIPs with exceptional molecular recognition capabilities for the template, functioning on a “lock-and-key” principle. When utilized as the sorbent in a solid-phase extraction format, MIPs form the basis of MISPE, enabling highly selective extraction and purification of the target from complex matrices. A key operational advantage of MIPs is their remarkable physicochemical stability, withstanding high temperatures, pressure, and exposure to acids, bases, metal ions, and organic solvents, which ensures robustness throughout the extraction process [48].

The high selectivity of MISPE makes it particularly valuable for isolating BaP from complex food samples. Pschenitza et al. [49] demonstrated this by developing a MISPE method coupled with enzyme-linked immunosorbent assay (ELISA) for the analysis of BaP in vegetable oils, reporting a detection limit of 0.61 μg/kg and recoveries of 63–114%. This approach effectively cleaned up the samples, with all measured BaP concentrations falling below the stringent EU limit of 2 μg/kg. This study marked the first report of a MISPE-ELISA combined method for BaP detection in edible oils, highlighting the practicality of MISPE as an efficient sample preparation tool. Beyond traditional SPE formats, the principle of molecular imprinting is also being successfully integrated into sensor technology to create highly selective detection platforms. For instance, a novel electrochemical sensor was fabricated by constructing a molecularly imprinted film on a complex substrate containing MoS_2_, peanut shell biochar, gold nanoparticles, and nitrogen-doped carbon dots. This sensor achieved a wide detection range (5 nM to 20 μM) and a low detection limit (1.5 nM) for BaP, demonstrating excellent stability, reproducibility, and selectivity in the analysis of edible oil samples [50]. This convergence of imprinting with sensor technology underscores the broad utility of molecular recognition strategies.

The primary strengths of MISPE lie in its superior selectivity, which directly translates to reduced matrix effects and improved method reliability [51]. It also generally offers straightforward operation, controllable costs, and good recovery rates with high precision. However, a critical challenge associated with MISPE is the potential for template leakage during the extraction process, where residual template molecules bleed from the polymer, leading to overestimation of the analyte concentration [52]. Furthermore, the synthesis of high-performance MIPs can be complex and time-consuming, often requiring optimization of monomers and cross-linkers. Future prospects for MISPE are promising and are likely to focus on addressing these limitations. Key directions include the development of novel imprinting strategies, such as the use of dummy templates (structurally similar but analytically distinct molecules) to eliminate the issue of template leakage entirely [53]. Efforts are also underway to synthesize MIPs with enhanced binding capacity and kinetics, and to seamlessly integrate MISPE columns into automated analytical systems, thereby increasing throughput and reproducibility for routine food safety monitoring.

### 2.2. Liquid–Liquid Extraction and Related Techniques

#### 2.2.1. Conventional Liquid–Liquid Extraction (LLE)

Conventional LLE is a foundational separation technique based on the “like dissolves like” principle, utilizing the differential partitioning of a target compound between two immiscible solvents, typically an aqueous phase and an organic phase [54]. The extraction efficiency is governed by the partition coefficient of the analyte. While LLE is operationally straightforward and low-cost, it has significant drawbacks, including the consumption of large volumes of organic solvents, tedious manual operation, and time-consuming processes [55]. These limitations, coupled with environmental concerns and potential health risks for operators, have hindered its application in modern analytical practices. Consequently, for the analysis of trace-level contaminants like BaP, there is a clear shift towards more efficient, miniaturized, and environmentally friendly sample preparation methods.

#### 2.2.2. Vortex-Assisted Liquid–Liquid Microextraction (VALLME)

As a miniaturized and improved alternative, vortex-assisted liquid–liquid microextraction introduces high-speed vortexing to drastically increase the interfacial contact area between the two phases, thereby enhancing mass transfer and extraction efficiency [56]. This approach significantly reduces organic solvent consumption while maintaining or improving performance. For instance, Mo et al. [57] applied VALLME combined with UPLC for the determination of BaP in virgin and refined camellia oils, achieving a low LOD of 0.2 μg/kg and recoveries between 81.0% and 97.0%. Their study also corroborated that BaP levels were higher in virgin oils compared to their refined counterparts. VALLME is thus recognized as a simple, rapid, and efficient technique well-suited for the routine analysis of BaP in fatty matrices. A primary limitation, however, is the potential for manual operation to become a bottleneck in high-throughput labs, and future developments could focus on its integration into automated sequential injection systems to further enhance reproducibility and efficiency [58].

### 2.3. Modern Microextraction and Integrated Approaches

#### 2.3.1. Supramolecular Solvent Microextraction (SUSME)

Supramolecular solvent microextraction utilizes nano-structured solvents that form through self-assembly processes, offering excellent extraction efficiency and clean-up capabilities for a wide range of analytes, making them ideal for complex matrices [59]. Wang et al. [60] established a method employing alkaline saponification combined with in situ SUSME for the selective extraction of BaP from edible oils, followed by HPLC-FLD detection. The method demonstrated good linearity, high recoveries (94–102%), and impressive sensitivity (LOD of 0.06 μg/kg). The supramolecular solvent effectively purified the sample by excluding saponifiable matter, showcasing how SUSME synchronizes extraction and purification into a single, efficient step. While highly effective, the tailored design of supramolecular solvents for specific applications can be complex. Therefore, the future of SUSME likely lies in the rational design of novel solvents with even greater selectivity for target analyte classes and the exploration of their compatibility with direct coupling to analytical instruments [61].

#### 2.3.2. QuEChERS

The QuEChERS (Quick, Easy, Cheap, Effective, Rugged, and Safe) approach is widely adopted for multi-residue analysis. Its core procedure involves an acetonitrile extraction assisted by salting-out, followed by a clean-up step using d-SPE to remove various co-extractives [62]. Its versatility is demonstrated in applications for BaP. Eslamizad et al. [63] developed a modified QuEChERS method for bread samples, achieving a low LOD of 0.3 µg/kg and recoveries of 95–120% by GC-MS. Similarly, Benny et al. [37] established a QuEChERS protocol suitable for PAHs in hydro-alcoholic plant extracts, confirming its utility for routine monitoring. The major strength of QuEChERS is its ability to provide a highly efficient and economical sample preparation solution. The main challenge lies in the need to optimize the d-SPE sorbent combination for different food matrices to ensure effective clean-up. Future trends involve the development of application-specific d-SPE kits and a stronger push towards full automation of the entire QuEChERS workflow to maximize its throughput and robustness in routine laboratories.

### 2.4. Gel Permeation Chromatography (GPC)

GPC, operating on the principle of molecular size exclusion, utilizes a porous stationary phase to efficiently separate high molecular weight interferents (e.g., lipids, pigments, proteins) from low molecular weight target analytes like BaP [64]. It is a high-throughput purification technique particularly advantageous for samples rich in macromolecular contaminants. The prominent benefits of GPC include effective lipid removal, significant reduction of matrix effects, and improved chromatographic performance. Furthermore, the process is highly amenable to automation, minimizing manual errors. However, its application is constrained by the relatively high cost of the instrumentation and the comparatively long analysis time per sample.

The utility of GPC for complex food matrices is well-demonstrated. An advanced GPC system was developed for the sensitive determination of EU-regulated PAHs, including BaP, in olive oil [65]. This innovative system, employing two columns and a switching valve, enabled direct clean-up without a preliminary extraction step and achieved a seven-fold increase in sample loading capacity (1 g vs. 0.15 g). This enhancement contributed to achieving impressively low detection limits for BaP (LOD 0.32 µg/kg), meeting stringent regulatory demands. While GPC is a powerful tool for lipid removal, its operational cost and time investment mean it is often best suited for laboratories with high sample throughput needs. The future of GPC likely involves integration with other purification techniques in multidimensional systems to achieve even higher specificity and the continued development of more efficient columns to reduce solvent consumption and analysis time.

The selection of an optimal sample preparation strategy is a critical decision, contingent upon the specific food matrix, the required sensitivity, and available laboratory resources. The challenge of lipid removal in fatty foods exemplifies the critical trade-offs between the major techniques discussed: GPC provides thorough, size-exclusive cleanup but with higher time and solvent costs; adsorption-based methods (e.g., SPE, d-SPE) offer greater speed and flexibility, yet require careful optimization to balance lipid removal efficiency with target analyte recovery. As evidenced throughout this section and as systematically compared in Table 2, each method presents a unique balance of selectivity, efficiency, and practicality.

## 3. Detection and Chromatographic Analysis Techniques

The accurate determination of BaP hinges not only on efficient sample preparation but also on the selection of an appropriate analytical technique dictated by the analyte’s intrinsic properties and regulatory requirements. This review critically evaluates the two established benchmark methods for BaP quantification: GC-MS and HPLC-FLD. This focus is grounded in the physicochemical nature of BaP and prevailing regulatory standards (e.g., EN 16619 [66]). For context, LC-MS/MS—while a powerful and indispensable tool for multi-residue analysis of polar or labile contaminants—presents specific challenges for the definitive quantification of stable, non-polar PAHs like BaP. These include lower ionization efficiency under common interfaces and heightened susceptibility to matrix effects, necessitating meticulous optimization and the mandatory use of isotope-labeled internal standards. Consequently, LC-MS/MS is less frequently the method of choice in BaP-specific regulatory methods but remains valuable for comprehensive PAH screening.

Following this evaluation of gold-standard methods, we examine emerging rapid and non-chromatographic techniques (e.g., sensors, immunoassays, spectroscopy) that offer advantages in speed and portability for screening purposes, acknowledging their complementary role alongside confirmatory laboratory methods.

### 3.1. Chromatographic Techniques for Separation and Quantification

Chromatographic methods form the backbone of regulatory analysis for BaP, providing the high separation power required to isolate the target from complex food matrices. The techniques discussed below share the common principle of separating components in a mixture before specific detection, thereby offering high selectivity, sensitivity, and robust quantitative capability.

#### 3.1.1. GC-MS

GC-MS is a robust analytical technique that capitalizes on the high separation efficiency of gas chromatography and the powerful identification capability of mass spectrometry, enabling the accurate qualitative and quantitative determination of target compounds. It is recognized as a reliable method for BaP analysis, particularly well-suited for handling complex matrices like lipids, owing to its rapid analysis speed, high resolution, and strong discriminatory power [67].

The applicability of GC-MS is well-documented in monitoring BaP in various food commodities. For instance, a method developed for fried foods demonstrated satisfactory performance with spiked recoveries ranging from 84.6% to 103.2% and relative standard deviations (RSDs) between 3.21% and 8.32% [68]. Beyond single-analyte detection, GC-MS, especially in the more selective tandem mass spectrometry (GC-MS/MS) mode, proves highly effective for the concurrent analysis of multiple PAHs. The method successfully detected 11 PAHs, including BaP, in shrimp samples, revealing distinct regional contamination. This GC-MS/MS analysis exhibited adequate linearity with a limit of quantification (LOQ) for BaP of 6.67 µg/kg [69]. Furthermore, GC-MS serves as a critical tool for human health risk assessment. Research on popular smoked meats and fish in Bangladesh quantified several PAHs via GC-MS, revealing that BaP concentrations (6.68–46.90 µg/kg) in smoked meats consistently exceeded the EU regulatory limit (5.0 µg/kg), flagging significant food safety concerns [70].

While GC-MS is a powerful technique for BaP analysis, several analytical considerations must be acknowledged. The high molecular weight and boiling point of BaP often necessitate elevated chromatographic temperatures, which can extend run times and increase wear on the GC system. A practical advantage of GC-MS for BaP analysis is that its chemical stability and non-polar nature typically obviate the need for a derivatization step. Ultimately, the accuracy of GC-MS determination is highly dependent on the efficiency of sample extraction and clean-up, which are critical for mitigating matrix effects and safeguarding instrument integrity. For definitive quantification, the use of stable isotope-labeled internal standards is indispensable. These standards co-extract and co-analyze with the native analyte, thereby compensating for both matrix-induced signal variations and analyte losses throughout the analytical process, which is critical for achieving high accuracy at trace levels. This isotope-dilution approach is the cornerstone of accurate quantification in mass spectrometry and is especially critical for LC-MS/MS, where matrix effects can be particularly severe and variable. As highlighted in seafood analysis, effective sample preparation directly influences the reliability of quantification, underscoring the necessity of matrix-specific method optimization and validation to ensure robust performance in complex food commodities [63].

#### 3.1.2. HPLC-FLD

HPLC-FLD remains a widely regarded benchmark technique for the determination of BaP, capitalizing on the compound’s inherent fluorescent properties. By meticulously optimizing the chromatographic conditions, including the chromatographic column and specific excitation/emission wavelengths, this method achieves exceptional selectivity and sensitivity for BaP, effectively minimizing interference from complex food matrices [71]. The widespread availability and relative affordability of HPLC-FLD instrumentation further consolidate its status as a routine and reliable analytical approach in many laboratories [72].

The robustness of HPLC-FLD is evidenced by its diverse applications in monitoring BaP across various food commodities, often in conjunction with innovative sample preparation strategies. For instance, it has been effectively employed to investigate the impact of processing conditions, revealing that thermally stressed foods, such as sesame oil produced via hot-pressing, contain significantly higher BaP levels compared to their cold-pressed counterparts, with concentrations positively correlating with roasting temperature and duration [73]. Methodological innovations often focus on streamlining the sample preparation. A study on fried and baked foods demonstrated that a supramolecular solvent (SUPRAS) extraction coupled with HPLC-FLD enabled rapid analysis (approx. 30 min per sample), achieving high sensitivity for BaP (LOD of 0.11 µg/kg) and satisfactory recoveries (89.86–100.01%) [74]. Similarly, for complex matrices like tea, both microwave-assisted and classical solid–liquid extraction have been successfully paired with HPLC-FLD for direct determination, bypassing tedious clean-up procedures while maintaining good accuracy [75]. Furthermore, the use of magnetic nanoparticles (Fe_3_O_4_@DA/GO) for BaP enrichment prior to HPLC-FLD analysis exemplifies a trend towards methods that offer rapid operation and high performance for the analysis of edible oils [76].

Notwithstanding its merits, a critical consideration for HPLC-FLD is its inherent dependence on a thorough chromatographic separation to accurately isolate BaP from other co-extracted fluorescent compounds, which can be particularly challenging in heavily contaminated samples containing structurally similar PAHs. Moreover, the technique’s performance is highly contingent on the efficacy of the preceding extraction and clean-up steps; any failure to remove interfering matrix components can lead to significant quantification errors. While advanced strategies like SUPRAS or magnetic solid-phase extraction simplify the process, they require meticulous optimization to mitigate potential matrix effects. Therefore, although HPLC-FLD is a powerful and established technique, its application must be critically evaluated and validated for each specific food matrix. Future developments are likely to focus on creating more robust and standardized hybrid protocols that seamlessly integrate efficient clean-up steps with HPLC-FLD determination, further solidifying its role as a cost-effective and reliable workhorse for routine BaP monitoring in food safety laboratories [77].

A critical consideration for both GC-MS and HPLC-FLD is the mitigation of matrix effects and accurate quantification. Co-extracted matrix components, such as lipids and pigments, can suppress or enhance the analyte signal, leading to quantification errors. The benchmark strategy to address this is the use of stable isotope-labeled internal standards (SIL-IS), such as ^13^C-labeled or deuterated BaP (e.g., d_12_-BaP). These standards are added prior to sample preparation, compensating for analyte losses throughout the workflow and correcting for matrix effects in mass spectrometric detection, thereby ensuring high quantitative accuracy and precision [78]. For HPLC-FLD, where SIL-IS cannot be used, meticulous chromatographic separation from interfering fluorescent compounds is paramount.

### 3.2. Emerging Rapid and Non-Chromatographic Techniques

In contrast to the laboratory-based chromatographic methods, this section covers techniques often designed for rapid screening, on-site analysis, or higher throughput. These methods frequently forego complex separation steps, instead relying on molecular recognition or unique physicochemical properties for detection. While they may not match the multi-residue confirmation power of chromatography, they address the growing need for speed and operational simplicity. Therefore, these techniques are best regarded as high-throughput presumptive screening tools. Their utility lies in rapidly identifying samples that require further analysis, with positive findings necessitating confirmation by definitive chromatographic methods such as GC-MS or LC-MS/MS. Their main methodological constraints include inherent risks of cross-reactivity (particularly for immunoassays), vulnerability to matrix-induced interferences that compromise accuracy, and the ongoing challenge of achieving robust validation across diverse food commodities.

#### 3.2.1. Sensor Technology

Sensor-based technologies, as emerging alternatives to conventional chromatographic methods, offer significant potential for the rapid and on-site screening of BaP in foods. These methods primarily encompass electrochemical and optical sensors, each with distinct operational mechanisms and application prospects [79].

Electrochemical sensors function by transducing electrical signals generated from redox reactions at a specifically modified electrode surface [80]. Their strengths typically include rapid response, high sensitivity, and operational stability. A significant advancement in this category is the development of a molecularly imprinted electrochemiluminescence (MIECL) sensor. This sophisticated platform integrated a covalent organic framework composite (COF-300-Au) supporting CsPbBr_3_ quantum dots as luminophores with a cross-linked polythiophene molecularly imprinted layer for specific BaP recognition. Leveraging a quenching mechanism, this sensor achieved an exceptionally low detection limit of 4.1 × 10^−15^ M (corresponding to approximately 0.001 µg/kg) alongside a wide linear range and satisfactory recoveries of 94.4–103.2% in edible oil, demonstrating remarkable stability and anti-interference capability [81]. Despite such impressive analytical performance, a critical barrier to practical implementation remains the vulnerability of the sensing interface to fouling and passivation in complex food matrices like oils, which can severely compromise sensor longevity and reliability. Consequently, while electrochemical sensors show extraordinary promise for rapid screening, their transition from proof-of-concept to routine application necessitates more extensive validation with real food samples and strategies to enhance interfacial robustness [82].

Optical sensors, on the other hand, detect analytes through their interaction with light, offering advantages such as rapid response and non-destructiveness. In BaP detection, surface-enhanced Raman spectroscopy (SERS) has garnered considerable attention due to its single-molecule-level sensitivity. A key challenge in SERS analysis of BaP is its weak affinity for conventional metal substrates. To address this, Zhang et al. [83] developed an SERS sensor utilizing a gold nanostar@reduced graphene oxide (AuNS@rGO) substrate. This design synergistically combined the superior adsorption and enrichment capability of rGO with the strong electromagnetic field enhancement from AuNS, enabling highly sensitive detection of BaP with an LOD as low as 0.0028 μg/kg and recoveries of 89.2–100.8% in spiked chicken samples. This study provides a valuable reference for achieving specific and highly sensitive optical sensing of trace BaP in complex food matrices. Despite such promising results, the widespread adoption of SERS faces significant challenges pertaining to the practical deployment of the substrates. The long-term stability, batch-to-batch reproducibility of the nanostructured substrates, and the competitive adsorption of interfering molecules in complex food samples continue to be major obstacles that require further resolution.

The future development of sensors for BaP detection will likely focus on creating more robust and selective recognition interfaces, integrating sample preparation steps seamlessly into sensor designs, and advancing reproducible nanofabrication techniques to ensure reliability. The ultimate goal remains the realization of cost-effective, user-friendly, and field-deployable sensors that can provide reliable quantitative or semi-quantitative data outside specialized laboratory settings, thereby transforming food safety monitoring paradigms.

#### 3.2.2. Immunoassays

##### Enzyme-Linked Immunosorbent Assay (ELISA)

ELISA operates on the principle of immobilized antigens or antibodies and enzyme-labeled conjugates, with quantitative or qualitative analysis achieved by measuring the color intensity from enzymatic substrate reactions [84]. The high catalytic efficiency of enzymes significantly amplifies the immunoassay signal, which grants this method exceptional sensitivity and makes it suitable for high-throughput screening. The performance of ELISA fundamentally depends on the quality and specificity of the antibody used. For instance, Pschenitza et al. [49] employed an SPE-ELISA approach for edible oils, achieving an LOD of 0.61 μg/kg. Xi et al. [85] developed an indirect competitive ELISA (ic-ELISA) kit based on a highly specific monoclonal antibody, with a half-maximal inhibition concentration (IC_50_) of 0.78 μg/kg and an LOD of 0.054 μg/kg, whose reliability was confirmed through 100% correlation with GC-MS results. Further demonstrating the method’s adaptability, Jeeno et al. [86] established an ic-ELISA using a polyclonal IgY antibody, achieving an LOD of 1.24 μg/kg in grilled pork samples, which meets the EU regulatory limit. A primary limitation of this technique, however, is the potential for antibody cross-reactivity with structurally similar PAHs. This was evidenced in the latter study, where the antibody exhibited substantial cross-reactivity (311.32%) with benzo[b]fluoranthene, a phenomenon that can lead to false positives or overestimation and necessitates confirmatory analysis by chromatographic methods. Despite this constraint, ELISA offers significant advantages, typically avoiding complex sample clean-up, requiring relatively low technical skill, and providing high throughput, which makes it ideal for large-scale screening. Consequently, future development should focus on generating antibodies with higher specificity through novel hapten design and immunization strategies, alongside the incorporation of more sophisticated sample preparation to minimize matrix effects in the most challenging food commodities [87,88].

##### Lateral Flow Immunoassay (LFIA)

LFIA enables rapid on-site detection by leveraging the specific antigen–antibody reaction on a nitrocellulose membrane, with signals generated from labels such as colloidal gold or fluorescent microspheres [89]. Compared to ELISA, LFIA is notably more user-friendly, requires no sophisticated instrumentation, and integrates purification within the chromatographic process, allowing for visual, real-time results. A significant advancement was demonstrated by Yuan et al. [90], who developed a multiplex signal LFIA using Prussian blue nanoparticles (PBNPs) to label monoclonal antibodies. The PBNPs’ peroxidase-like activity catalyzed a substrate to amplify the signal, enabling simultaneous colorimetric, photothermal, and catalytic colorimetric detection within 14 min. This multi-modal approach achieved recoveries of 86–105% in vegetable oils, with LODs between 0.057 and 0.106 μg/kg and a wide linear range, representing a sensitivity improvement of 175 to 285-fold over traditional colorimetric LFIAs. Despite its great promise for field-based screening, LFIA is inherently a semi-quantitative or quantitative screening tool. Its quantitative accuracy and robustness can be susceptible to matrix interference, and like ELISA, it may suffer from cross-reactivity issues. Therefore, its primary application remains rapid on-site screening, with positive results requiring laboratory confirmation.

#### 3.2.3. Spectroscopic and Other Techniques

##### Molecular Fluorescence Spectroscopy

Molecular fluorescence spectroscopy offers attractive alternatives for the rapid screening of BaP by leveraging its distinct fluorescent signature, often with minimal sample preparation. Constant-wavelength synchronous fluorescence (CWSF) enhances selectivity by scanning both the excitation and emission monochromators while maintaining a fixed wavelength difference (Δλ), effectively narrowing spectral bands. Soares et al. [91] developed a CWSF method for barley malt that required only a simplified acetonitrile extraction, achieving LOD of 0.24 μg/kg and a linear range of 0.5–3.0 μg/kg for BaP. With recoveries of 97.9–114.6% and an RSD below 10%, the complete analysis was accomplished within 2.5 h. While this technique successfully bypasses complex purification, its primary limitation is potential spectral overlap from other PAHs, which may necessitate the use of matrix-matched calibration or chromatographic confirmation for definitive results.

Direct fluorescence spectroscopy further reduces reliance on extensive sample pretreatment by targeting BaP’s characteristic emission peak (around 405 nm), capitalizing on its minimal spectral overlap with matrix components in certain commodities. Orfanakis et al. [92] demonstrated this approach for extra virgin olive oil by combining excitation–emission matrix (EEM) fluorescence with a partial least squares (PLS) regression model, achieving an LOD of 0.5 μg/kg without any pre-extraction. This method is notably cost-effective, simple, and rapid. However, its applicability is constrained by difficulties in simultaneously quantifying multiple PAHs, as specificity is heavily reliant on the robustness of the chemometric model. Performance can also be significantly compromised in highly complex or heterogeneous matrices, and it typically requires a laboratory-grade spectrofluorometer, limiting its use for field analysis.

##### Terahertz Spectroscopy

Terahertz (THz) spectroscopy represents an emerging, nearly non-destructive technique that probes characteristic vibrational absorptions of molecules within the 0.1–2.5 THz frequency range, coupled with chemometrics for quantification. Liu et al. [93] pioneered its application for the rapid quantification of BaP in soybean oil, developing a model that achieved a recognition accuracy of 96.19% across a concentration range of 0–20 μg/kg. This technology demonstrates considerable potential for non-destructive, rapid, and even online monitoring. Nevertheless, a significant challenge lies in the weak THz absorption signals of BaP at low concentrations (particularly below 6 μg/kg), which are easily masked by the dominant signal from the oil matrix. Consequently, the technique’s success is highly dependent on sophisticated algorithms for signal resolution and ongoing model optimization, currently restricting its use to research settings. Future advancements in high-sensitivity THz sources, detectors, and tailored multivariate analysis methods are crucial to unlocking their practical application in routine food safety control [94].

##### Emerging Rapid and Non-Chromatographic Techniques

Matrix-assisted laser desorption/ionization time-of-flight mass spectrometry (MALDI-TOF-MS) represents a soft ionization mass spectrometry technique characterized by its high throughput and exceptional sensitivity. A notable advancement was demonstrated by Wang et al. [95], who established a rapid method for BaP detection in edible oils using a metal–organic framework, MIL-101(Fe), as a novel matrix for MALDI-TOF-MS. Under optimized conditions, this approach achieved an impressive detection limit of 0.1 μg/kg with an analysis time of merely one minute per sample. When evaluated in complex matrices such as sesame and linseed oils, the method delivered satisfactory recoveries ranging from 80.0% to 114.8%, albeit with relative standard deviations (3.9–13.7%) that indicate room for improvement in precision. The technique offers compelling advantages, including minimal sample consumption (1 µL), exceptional speed, and a wide linear range, positioning it as a powerful tool for high-throughput rapid screening. However, its precision typically falls short of that achieved by more established chromatographic–mass spectrometric hyphenated techniques like GC-MS or LC-MS/MS. This limitation, often attributed to inherent signal heterogeneity in MALDI, alongside challenges related to matrix effects and quantitative reproducibility, currently restricts its primary application to semi-quantitative screening rather than definitive confirmatory analysis. Future prospects for MALDI-TOF-MS in BaP analysis are promising and hinge on the continued development of more homogeneous and efficient matrix materials, such as advanced nanomaterials and covalent organic frameworks, to improve shot-to-shot reproducibility [96]. Furthermore, the integration of internal standards designed specifically for MALDI and the advancement of robust calibration strategies are critical research directions to enhance its quantitative capability, potentially enabling its transition from a rapid screening tool to a reliable quantitative methodology [97].

A systematic comparison of the operational characteristics, performance, and challenges of the major extraction and detection techniques discussed is provided in Table 2 and Table 3, respectively. As detailed in Table 3, which offers a comprehensive overview of the detection methods, this comparative overview underscores that there is no universal ‘best’ method; rather, the choice is a trade-off dependent on the specific analytical requirements, such as the required sensitivity, sample throughput, matrix complexity, and the need for portability or confirmatory analysis [98].

## 4. Current Challenges and Future Perspectives

Despite substantial progress, the accurate and efficient determination of BaP in food continues to face several interconnected challenges. A primary obstacle remains the inherent trade-off between analytical throughput and the stringent requirements for sensitivity and accuracy, particularly when dealing with complex matrices such as edible oils, spices, and processed meat products. While modern approaches like QuEChERS and emerging sensor technologies offer significantly enhanced processing speed, their performance can be severely compromised by substantial matrix effects without meticulous, matrix-specific optimization protocols [100]. Another critical challenge is the translational gap between laboratory innovations in material science and their practical application. Although advanced materials, including nanomaterials, metal–organic frameworks, and MIPs, demonstrate exceptional performance in research settings, concerns regarding cost-effective mass production, long-term stability, and batch-to-batch reproducibility continue to hinder their widespread adoption in routine quality control laboratories [101]. Furthermore, the regulatory acceptance of rapid screening methods, such as LFIAs, SERS, and electrochemical sensors, remains a significant barrier. These techniques, despite their clear potential for on-site monitoring, require extensive validation and standardization before they can be reliably integrated into official surveillance programs.

Looking forward, the next wave of innovation is likely to focus on the convergence of advanced materials, miniaturization, and data science to create smarter, more connected analytical systems. The development of multifunctional and stimuli-responsive materials, such as advanced chemically modified electrodes and tailored SERS substrates, will be a primary direction, enabling more selective capture and enhanced detection of BaP directly in complex matrices [102,103]. The integration of these smart sensing interfaces with microfluidic and lab-on-a-chip platforms paves the way for fully automated “sample-in-answer-out” systems, which promise to minimize human error and deliver laboratory-grade analysis at the point of need. Another pivotal area for development is the growing application of machine learning (ML) and artificial intelligence (AI) to revolutionize data processing for rapid screening techniques. For example, ML algorithms such as convolutional neural networks are being employed to deconvolute the complex spectral signatures from techniques like SERS, demonstrating the potential to specifically identify target analytes within complex spectral backgrounds [104]. Similarly, the development of advanced, data-rich sensing platforms provides the essential foundation for subsequent AI-driven analysis, which can enhance detection accuracy and even predict contamination sources. Beyond data analysis, ML models also show promise in accelerating the design of novel sensing materials by predicting molecular interactions [105]. The key challenges in this novel area include generating large, high-quality datasets for model training, ensuring the robustness and regulatory acceptance of these models, and their seamless integration into user-friendly platforms. These computational tools can help deconvolute complex signals from techniques like SERS or sensor arrays, compensating for matrix effects and improving quantitative accuracy, which is crucial for building confidence in these methods [103]. Beyond these technological refinements, a strategic paradigm shift is anticipated, moving beyond BaP as a single marker toward holistic monitoring strategies. This involves utilizing non-targeted screening via high-resolution mass spectrometry to comprehensively profile PAH mixtures, coupled with effect-directed analysis to assess the overall toxicological potency of a sample. Consequently, achieving these goals will require intensified international collaboration for method standardization, which is essential for validating, regulating, and globally implementing these next-generation solutions, thereby establishing a more robust and comprehensive framework for protecting public health from BaP and related contaminants in food [106].

## 5. Conclusions

This review has systematically delineated the current landscape of analytical techniques for BaP in food, with a comprehensive span across advancements in both sample preparation and detection technologies. The field has demonstrably evolved from conventional, solvent-intensive methods toward modern micro-extraction and solid-phase techniques, aligning with the principles of green chemistry and high-throughput analysis. Concurrently, the detection arsenal has expanded beyond the established dominance of chromatographic methods to include a promising suite of rapid sensors and immunoassays.

A critical synthesis of these methodologies reveals that the optimal choice is inherently a fit-for-purpose decision. Chromatographic techniques, namely GC-MS and HPLC-FLD, remain the indispensable benchmark for confirmatory analysis and regulatory compliance due to their unmatched sensitivity and specificity. However, their operational demands limit utility for rapid screening. In this domain, immunological and sensor technologies hold transformative potential for on-site and high-throughput monitoring, pending the resolution of key challenges related to specificity, robustness, and matrix interference, as outlined in this review.

Ultimately, the path forward hinges on the strategic convergence of advanced materials, miniaturization, and data science, as explored in the perspectives section. Championing the development of intelligent, integrated, and automated “sample-in-answer-out” platforms constitutes the next frontier. The successful development and adoption of such systems, which are capable of moving beyond BaP as a single marker to assess comprehensive mixture risk, will be paramount for building a resilient, proactive, and globally harmonized framework for food safety, thereby strengthening public health protection against current and future chemical hazards.

## Figures and Tables

**Table 1:** Selected examples of maximum levels for benzo[a]pyrene in various food categories as regulated by China, the European Union, and the Codex Alimentarius Commission.

Country/Organization	Food Category	BaP (μg/kg)	Reference
China	Oils and fats	10.0	[29]
	Grains and products	5.0	
	Meat and products	5.0	
	Aquatic products	5.0	
European Union	Oils and fats	2.0	[30]
CAC	Edible oils	5.0	[31]

This table provides a comparative overview of selected key food categories and is not exhaustive. Numerous other commodity-specific MLs exist (e.g., for smoked meats and food supplements in the EU). Refer to the official regulations [29,30,31] for complete details.

**Table 2:** Comparison of extraction and purification techniques for BaP in foods.

Technique	Principle	Key Procedural Steps	Key Advantages	Key Limitations/Challenges	Refs.
Conventional SPE	Selective adsorption on solid sorbent	Condition sorbent Load sample Wash interferents Elute target	High selectivity, ease of automation, good reproducibility	Limited sorbent universality, high consumable cost	[36,37]
MSPE	Adsorption on functionalized magnetic nanoparticles, separated by magnet	Disperse MNPs in sample Magnetic separation Elute analyte from MNPs	Rapid operation, minimal solvent use, high efficiency	Susceptibility to matrix fouling, scalability and reproducibility of adsorbents	[40]
d-SPE	Sorbent dispersed in extract for cleanup	Add sorbent to extract Vortex mixing Centrifugation Collect purified supernatant	Simple, fast, low solvent consumption, high throughput	Potential need for customized sorbent mixtures, manual centrifugation step	[44,45]
MISPE	Selective recognition by molecularly imprinted polymers	Load sample Wash to remove non-specifically bound compounds Elute target with selective solvent	Exceptional selectivity, reduced matrix effects, robust	Template leakage risk, complex and time-consuming polymer synthesis	[49,50]
VALLME	Vortex-assisted partitioning between immiscible solvents	Mix sample with micro-volume of extraction solvent Vortex to form emulsion Centrifuge for phase separation Collect extraction phase	Rapid, low solvent consumption, high extraction efficiency	Manual operation bottleneck, requires automation for high reproducibility	[57]
SUSME	Extraction with nano-structured supramolecular solvents	Form or add supramolecular solvent Vortex mix Centrifuge to induce coacervation Collect solvent-rich phase	High efficiency, integrates extraction and purification, green	Complex solvent design for specific applications	[60]
QuEChERS	Acetonitrile extraction & d-SPE cleanup	Extract with acetonitrile and salt-out Collect acetonitrile phase Clean-up using d-SPE sorbents Collect final extract	Rapid, cost-effective, rugged, high throughput	Requires matrix-specific optimization of d-SPE sorbents	[37,63]
GPC	Size-exclusion separation of macromolecules	Load sample extract Elute with solvent; collect target analyte fraction Concentrate fraction for analysis	Excellent lipid removal, high automation, reduced matrix effects	High instrument cost, long analysis time	[65]

**Table 3:** Comprehensive comparison of analytical methods for the determination of BaP in foods.

Method Category	Target Analytes	Application	LOD for BaP (μg/kg)	Key Advantages	Key Limitations/Challenges	Refs.
GC-MS(/MS)	Multiple PAHs (including BaP)	Quantitative	~0.1–0.3	High sensitivity and confirmatory power; suitable for multiresidue analysis.	Requires extensive sample preparation; high instrument cost.	[68,69,70]
HPLC-FLD	BaP (or a few PAHs)	Quantitative	~0.1–0.2	High selectivity for BaP; cost-effective; widely available.	Requires effective separation and cleanup; time-consuming.	[73,74,75,76]
Electrochemical Sensors	BaP	Screening	~0.001	High sensitivity; rapid response; potential for portability.	Susceptible to matrix fouling; requires stability validation.	[81]
Optical Sensors (SERS)	BaP	Screening	~0.003	Exceptional sensitivity; provides molecular fingerprint.	Substrate reproducibility and stability are challenges.	[83]
ELISA	BaP (may cross-react)	Screening	~0.6–1.2	High throughput; excellent for screening; minimal sample cleanup needed.	Cross-reactivity risk; typically semi-quantitative.	[49,85,86]
LFIA	BaP	Screening	~0.05–0.1	Ultra-fast; on-site; user-friendly; visual readout.	Semi-quantitative; susceptible to matrix effects.	[90]
Fluorescence Spectroscopy	BaP	Screening	~0.2–0.5	Rapid; minimal sample preparation; cost-effective.	Spectral overlap requires chemometrics.	[91,92]
MALDI-TOF-MS	BaP	Semi-Quantitative	~0.1	Ultra-fast analysis; high throughput; minimal sample prep.	Lower quantitative precision; matrix effects.	[95]

Reported LODs are approximate ranges or typical values in µg/kg, summarizing data from the cited references. For reference, the limits of detection stipulated by key international standard methods for the core confirmatory techniques are 0.30 µg/kg for GC-MS (EN 16619:2015 [66]) and 0.10 µg/kg for HPLC-FLD (ISO 15302:2017 [99]).

## Data Availability

No new data were created or analyzed in this study. Data sharing is not applicable to this article.

## References

[1] Mansour S.T., Ibrahim H., Zhang J., Farag M.A. (2025). Extraction and analytical approaches for the determination of post-food processing major carcinogens: A comprehensive review towards healthier processed food. Food Chem..

[2] Yin J.-j., Jiang X.-m., Nie Y., Zhong W., Zhang X.-h., Gao P., He D.-p. (2025). A comprehensive review on the formation and mitigation of polycyclic aromatic hydrocarbons (PAH4) in edible oils: From oilseeds to oils. Curr. Res. Food Sci..

[3] Cao Y., Zhao Q., Geng Y., Li Y., Huang J., Tian S., Ning P. (2021). Interfacial interaction between benzo[a]pyrene and pulmonary surfactant: Adverse effects on lung health. Environ. Pollut..

[4] Li X., Wang R., Lan X., Kong W., Zhu L., Xu W. (2026). Portable and in situ rapid detection of benzo[a]pyrene: A review. Food Chem..

[5] European Food Safety Authority (2008). Polycyclic Aromatic Hydrocarbons in Food—Scientific Opinion of the Panel on Contaminants in the Food Chain. EFSA J..

[6] Li W., Wu S. (2023). Challenges of halogenated polycyclic aromatic hydrocarbons in foods: Occurrence, risk, and formation. Trends Food Sci. Technol..

[7] Domingo J.L., Nadal M. (2015). Human dietary exposure to polycyclic aromatic hydrocarbons: A review of the scientific literature. Food Chem. Toxicol..

[8] Darwish W.S., Chiba H., El-Ghareeb W.R., Elhelaly A.E., Hui S.-P. (2019). Determination of polycyclic aromatic hydrocarbon content in heat-treated meat retailed in Egypt: Health risk assessment, benzo[a]pyrene induced mutagenicity and oxidative stress in human colon (CaCo-2) cells and protection using rosmarinic and ascorbic acids. Food Chem..

[9] Dobaradaran S., Schmidt T.C., Lorenzo-Parodi N., Kaziur-Cegla W., Jochmann M.A., Nabipour I., Lutze H.V., Telgheder U. (2020). Polycyclic aromatic hydrocarbons (PAHs) leachates from cigarette butts into water. Environ. Pollut..

[10] Su C., Zheng D., Zhang H., Liang R. (2023). The past 40 years’ assessment of urban-rural differences in Benzo[a]pyrene contamination and human health risk in coastal China. Sci. Total Environ..

[11] Acarer Arat S. (2024). A review on cigarette butts: Environmental abundance, characterization, and toxic pollutants released into water from cigarette butts. Sci. Total Environ..

[12] Wang J., Su X., Zhang C., Han Z., Wang M. (2025). Biodegradation of Benzo(a)pyrene in Contaminated Soil: Plant and Microorganism Contributions from Isotope Tracing. Toxics.

[13] Kowalska J., Stanisławek M., Latoch A., Marzec A., Galus S., Kowalska H., Ciecierska M. (2025). Polycyclic Aromatic Hydrocarbons in Polish Traditionally and Industrially Smoked Meats as an Element of Monitoring and PAH Reduction Strategies. Foods.

[14] Aresta A.M., Zambonin C. (2023). Determination of Polycyclic Aromatic Hydrocarbons (PAHs) in Coffee Samples by DI-SPME-GC/MS. Food Anal. Methods.

[15] Chen X., Liao Y., Lin B., He X., Li S., Zhong C., Li S., Zhou Y., Fan L. (2024). The Concentration of Benzo[a]pyrene in Food Cooked by Air Fryer and Oven: A Comparison Study. Toxics.

[16] Semanová J., Skláršová B., Šimon P., Šimko P. (2016). Elimination of polycyclic aromatic hydrocarbons from smoked sausages by migration into polyethylene packaging. Food Chem..

[17] Guerreiro T.M., de Oliveira D.N., Melo C.F.O.R., de Oliveira Lima E., Catharino R.R. (2018). Migration from plastic packaging into meat. Food Res. Int..

[18] Shi K., Feng X., Liu C., Liang J., Luo J. (2024). Combating regional air pollution significantly enhance the photodegradation of atmospheric benzo(a)pyrene. Sci. Total Environ..

[19] Zhu Z., Sun L., Qin Q., Sun Y., Yang S., Wang J., Yang Y., Gao G., Xue Y. (2024). The Adsorption Process and Mechanism of Benzo[a]pyrene in Agricultural Soil Mediated by Microplastics. Toxics.

[20] Defois C., Ratel J., Denis S., Batut B., Beugnot R., Peyretaillade E., Engel E., Peyret P. (2017). Environmental Pollutant Benzo[a]Pyrene Impacts the Volatile Metabolome and Transcriptome of the Human Gut Microbiota. Front. Microbiol..

[21] World Health Organization, International Agency for Research on Cancer (2010). Some non-heterocyclic polycyclic aromatic hydrocarbons and some related exposures. IARC Monographs on the Evaluation of Carcinogenic Risks to Humans.

[22] Bukowska B., Mokra K., Michałowicz J. (2022). Benzo[a]pyrene—Environmental Occurrence, Human Exposure, and Mechanisms of Toxicity. Int. J. Mol. Sci..

[23] Gan Y., Zhang X., Cai P., Zhao L., Liu K., Wang H., Xu D. (2024). The Role of Oxidative Stress and DNA Hydroxymethylation in the Pathogenesis of Benzo[a]pyrene-Impaired Reproductive Function in Male Mice. Environ. Toxicol..

[24] Mauliasari I.R., Lee H.J., Koo S.Y., Hitayezu E., Kieu A.N., Lee S.-M., Cha K.H. (2024). Benzo(a)pyrene and Gut Microbiome Crosstalk: Health Risk Implications. Toxics.

[25] Duan J., Li H., Wang Y., Ji Y., Chen C., Feng C., Zhang W. (2023). Benzo[a]pyrene and a high-fat diet induce aortic injury and promote low-density lipoprotein accumulation in the endothelium. Ecotoxicol. Environ. Saf..

[26] Zhang Z., Zhang W., Wang H., Chen H., Wang H., Yu Y., Shen D., Pi M., Wu Y., Luo M. (2025). Immunosuppressive role of benzo[a]pyrene exposure in prostate cancer progression. J. Environ. Sci..

[27] Stagaman K., Alexiev A., Sieler M.J., Hammer A., Kasschau K.D., Truong L., Tanguay R.L., Sharpton T.J. (2024). The zebrafish gut microbiome influences benzo[a]pyrene developmental neurobehavioral toxicity. Sci. Rep..

[28] Labib S., Guo C.H., Williams A., Yauk C.L., White P.A., Halappanavar S. (2013). Toxicogenomic outcomes predictive of forestomach carcinogenesis following exposure to benzo(a)pyrene: Relevance to human cancer risk. Toxicol. Appl. Pharmacol..

[29] (2017). National Food Safety Standard—Maximum Levels of Contaminants in Foods. https://wjw.nmg.gov.cn/zfxxgk/fdzzgknr/hybz/spbz/202111/t20211108_1925087.html.

[30] Commission Regulation (EC) (2011). No. 835/2011 of 19 August 2011 Amending Regulation (EC) No. 1881/2006 as Regards Maximum Levels for Polycyclic Aromatic Hydrocarbons in Foodstuffs.

[31] (1995). General Standard for Contaminants and Toxins in Food and Feed. https://www.fao.org/fao-who-codexalimentarius.

[32] (2016). National Food Safety Standard—Determination of Benzo[a]pyrene in Foods.

[33] (2019). Rapid detection of benzo[a]pyrene in edible oils—Colloidal gold immunochromatography.

[34] Hennion M.-C. (1999). Solid-phase extraction: Method development, sorbents, and coupling with liquid chromatography. J. Chromatogr. A.

[35] Yuan D., Zhang L., Ma F., Li P. (2022). Simultaneous Determination of Aflatoxins and Benzo(a)pyrene in Vegetable Oils Using Humic Acid-Bonded Silica SPE HPLC–PHRED–FLD. Toxins.

[36] Hu M., Zhu M., Xin L., Zhang G., Wu S., Hu X., Gong D. (2021). Change of benzo(a)pyrene during frying and its groove binding to calf thymus DNA. Food Chem..

[37] Benny A.M., Benny A., Aravind E.S. (2021). Determination of PAH Benzo[a]anthracene, Chrysene, Benzo[b]flouranthene and Benzo[a]pyrene in Hydro Alcoholic Herbal Extracts with Fluorescence Detector Using Solid-Phase Extraction. J. Chromatogr. Sci..

[38] Choodum A., Lamthornkit N., Boonkanon C., Taweekarn T., Phatthanawiwat K., Sriprom W., Limsakul W., Chuenchom L., Wongniramaikul W. (2021). Greener Monolithic Solid Phase Extraction Biosorbent Based on Calcium Cross-Linked Starch Cryogel Composite Graphene Oxide Nanoparticles for Benzo(a)pyrene Analysis. Molecules.

[39] He M., Chen Z., Xu C., Chen B., Hu B. (2021). Magnetic nanomaterials as sorbents for trace elements analysis in environmental and biological samples. Talanta.

[40] Pan Y., Deng Z., Chen Y., Zhang W., Yang Z., Zhao W., Zhang S. (2017). Determination of benzo[a]pyrene in smoked foods by high-performance liquid chromatography based on magnetic solid phase extraction. Anal. Methods.

[41] Wang N., Zhou X., Cui B. (2023). Recent advances and applications of magnetic covalent organic frameworks in food analysis. J. Chromatogr. A.

[42] Chen R., Qiao X., Liu F. (2022). Ionic liquid-based magnetic nanoparticles for magnetic dispersive solid-phase extraction: A review. Anal. Chim. Acta.

[43] Belarbi S., Vivier M., Zaghouani W., De Sloovere A., Agasse V., Cardinael P. (2021). Comparison of Different d-SPE Sorbent Performances Based on Quick, Easy, Cheap, Effective, Rugged, and Safe (QuEChERS) Methodology for Multiresidue Pesticide Analyses in Rapeseeds. Molecules.

[44] Lv Z., Yang C., Pang Y., Xie W., Shen X. (2019). Dispersive solid-phase extraction using the metal–organic framework MIL-101(Cr) for determination of benzo(a)pyrene in edible oil. Anal. Methods.

[45] Wang Q., Ren X., Tang S.-F. (2024). Three-dimensional hierarchical porous biochar as an efficient adsorbent for simultaneous extraction of ametryn and benzo[a]pyrene from tea beverages. J. Mol. Struct..

[46] Peter M., Müller C. (2024). Systematic Comparison of Extract Clean-Up with Currently Used Sorbents for Dispersive Solid-Phase Extraction. Molecules.

[47] Martins R.O., Cardoso A.T., Borsatto J.V., Lanças F.M. (2025). Advances in green carbon-based biosorbents: From conventional to miniaturized sample preparation strategies. Talanta.

[48] Eissa M.S., Imam M.S., AbdElrahman M., Ghoneim M.M., Abdullah M., Bayram R., Ali H.M., Abdelwahab N.S., Gamal M. (2024). Magnetic molecularly imprinted polymers and carbon dots molecularly imprinted polymers for green micro-extraction and analysis of pharmaceuticals in a variety of matrices. Microchem. J..

[49] Pschenitza M., Hackenberg R., Niessner R., Knopp D. (2014). Analysis of Benzo[a]pyrene in Vegetable Oils Using Molecularly Imprinted Solid Phase Extraction (MISPE) Coupled with Enzyme-Linked Immunosorbent Assay (ELISA). Sensors.

[50] Chi H., Li Y., Liu G. (2022). A molecularly imprinted electrochemical sensor based on a MoS_2_/peanut shell carbon complex coated with AuNPs and nitrogen-doped carbon dots for selective and rapid detection of benzo(a)pyrene. Int. J. Food Sci. Technol..

[51] Hu T., Chen R., Wang Q., He C., Liu S. (2021). Recent advances and applications of molecularly imprinted polymers in solid-phase extraction for real sample analysis. J. Sep. Sci..

[52] Nicholls I.A., Golker K., Wiklander J.G. (2026). The evolution of molecular dynamics as a tool for the study and development of molecularly imprinted materials—Status quo, quo vadis?. TrAC Trends in Anal. Chem..

[53] Wang S., Hu X., Wu W., Wang D., Li P., Zhang Z. (2024). Dual-template magnetic molecularly imprinted polymers for selective extraction and sensitive detection of aflatoxin B1 and benzo(α)pyrene in environmental water and edible oil. Food Chem..

[54] Pramanik S., Islam A.S.M., Ghosh I., Ghosh P. (2024). Supramolecular chemistry of liquid–liquid extraction. Chem. Sci..

[55] Shin J.m., Choi S.-J., Park Y.h., Kwak B., Moon S.H., Yoon Y.T., Jo S.A., Yi H., Kim S.j., Park S.K. (2022). Comparison of QuEChERS and Liquid–Liquid extraction methods for the simultaneous analysis of pesticide residues using LC-MS/MS. Food Control.

[56] Abu-Bakar N.-B., Makahleh A., Saad B. (2014). Vortex-assisted liquid–liquid microextraction coupled with high performance liquid chromatography for the determination of furfurals and patulin in fruit juices. Talanta.

[57] Mo R., Zhang Y., Ni Z., Tang F. (2017). Determination of benzo[a]pyrene in camellia oil via vortex-assisted extraction using the UPLC-FLD method. Food Sci. Biotechnol..

[58] Zaibi, Shah Z.A., Ullah R., Ali E.A., Toloza C.A.T., Hauser-Davis R.A., Muhammad U., Khan S. (2023). Ammonia Mediated Silver Nanoparticles Based Detection of Bisphenol A, an Endocrine Disruptor, in Water Samples after Vortex-Assisted Liquid–Liquid Microextraction. Chemosensors.

[59] Dalmaz A., Sivrikaya Özak S. (2023). Environmentally-friendly supramolecular solvent microextraction method for rapid identification of Sudan I–IV from food and beverages. Food Chem..

[60] Wang J., Liu L., Shi L., Yi T., Wen Y., Wang J., Liu S. (2017). Determination of benzo[a]pyrene in edible oils using phase-transfer-catalyst-assisted saponification and supramolecular solvent microextraction coupled to HPLC with fluorescence detection. J. Sep. Sci..

[61] Algar L., Sicilia M.D., Rubio S. (2023). Ribbon-shaped supramolecular solvents: Synthesis, characterization and potential for making greener the microextraction of water organic pollutants. Talanta.

[62] Elattar R.H., Kamal El-Deen A. (2024). Porous material-based QuEChERS: Exploring new horizons in sample preparation. TrAC Trends Anal Chem..

[63] Eslamizad S., Yazdanpanah H., Javidnia K., Sadeghi R., Bayat M., Shahabipour S., Khalighian N., Kobarfard F. (2016). Validation of an analytical method for determination of benzo[a]pyrene bread using QuEChERS method by GC-MS. Iran. J. Pharm. Res..

[64] Boborodea A., O’Donohue S. (2016). Low solvent consumption gel permeation chromatography method. Int. J. Polym. Anal. Charact..

[65] Cotugno P., Massari F., Aresta A., Zambonin C., Ragni R., Monks K., Avagyan L., Böttcher J. (2021). Advanced Gel Permeation Chromatography system with increased loading capacity: Polycyclic aromatic hydrocarbons detection in olive oil as a case of study. J. Chromatogr. A.

[66] (2015). Food analysis—Determination of benzo[a]pyrene, benz[a]anthracene, chrysene and benzo[b]fluoranthene in foodstuffs by gas chromatography mass spectrometry (GC-MS).

[67] Xu M.-L., Gao Y., Wang X., Han X.X., Zhao B. (2021). Comprehensive Strategy for Sample Preparation for the Analysis of Food Contaminants and Residues by GC–MS/MS: A Review of Recent Research Trends. Foods.

[68] Liu H., Shao J., Lin T., Li Q. (2013). Detection of Benzo[a]pyrene in Fried Food by Ultrasound-Assisted Matrix Solid-Phase Dispersion and Isotope Dilution GC–MS. Chromatographia.

[69] Arias J.L.d.O., Meireles A.C.N., Kulzer J., de Oliveira L.T., Valle S.L.d., Borba V.S.d., Kupski L., Barbosa S.C., Primel E.G. (2024). A vortex-assisted MSPD method for the extraction of Polycyclic Aromatic Hydrocarbons from shrimp with determination by GC-MS/MS. J. Chromatogr. A.

[70] Tareq A.R.M., Afrin S., Hossen M.S., Hashi A.S., Quraishi S.B., Nahar Q., Begum R., Ullah A.K.M.A. (2022). Gas Chromatography–Mass Spectrometric (GC-MS) Determination of Polycyclic Aromatic Hydrocarbons in Smoked Meat and Fish Ingested by Bangladeshi People and Human Health Risk Assessment. Polycyclic Aromat. Compd..

[71] Gazioglu I., Tekkeli S.E.K. (2017). Development and validation of a HPLC method for the determination of benzo(a)pyrene in human breast milk. Food Sci. Biotechnol..

[72] Bereda G. (2025). Emerging technologies and strategies for food contaminant detection and control: A global narrative review. J. Food Compos. Anal..

[73] Cheng W., Liu G., Wang X., Liu X., Liu B. (2015). Formation of Benzo(a)pyrene in Sesame Seeds During the Roasting Process for Production of Sesame Seed Oil. J. Am. Oil Chem. Soc..

[74] Yang S.-d., Tang T., Tan Y.-m., Wang F.-y., Zhang W.-b., Li T., Xia M.-z. (2019). Determination of benzo(a)pyrene in fried and baked foods by HPLC combined with vesicular coacervative supramolecular solvent extraction. J. Food Sci. Technol..

[75] Aygun S.F., Dikbas C., Tembo Z.N. (2025). Determination of carcinogenic benzo(a)pyrene in heat treated black tea samples from Turkey by using HPLC-fluorescence detection system. J. Food Sci. Technol..

[76] Li X., Sun C.-L., Xu Y., Shan S.-H., Zheng H., Guo X.-L., Hu J.-N. (2022). Construction of novel magnetic nanoparticles for enrichment of benzo(α)pyrene from edible oils followed by HPLC determination. Food Chem..

[77] Tsiasioti A., Tzanavaras P.D. (2024). High performance liquid chromatography coupled with post-column derivatization methods in food analysis: Chemistries and applications in the last two decades. Food Chem..

[78] Cortese M., Gigliobianco M.R., Magnoni F., Censi R., Di Martino P. (2020). Compensate for or Minimize Matrix Effects? Strategies for Overcoming Matrix Effects in Liquid Chromatography-Mass Spectrometry Technique: A Tutorial Review. Molecules.

[79] Weston M., Geng S., Chandrawati R. (2021). Food Sensors: Challenges and Opportunities. Adv. Mater. Technol..

[80] Hernaez M. (2020). Applications of Graphene-Based Materials in Sensors. Sensors.

[81] Chi H., Wang L., Wang S., Liu G. (2023). An electrochemiluminescence sensor based on CsPbBr3 -zquantum dots and poly (3-thiophene acetic acid) cross-linked nanogold imprinted layer for the determination of benzo(a)pyrene in edible oils. Food Chem..

[82] Zhou S., Liu C., Lin J., Zhu Z., Hu B., Wu L. (2022). Towards Development of Molecularly Imprinted Electrochemical Sensors for Food and Drug Safety: Progress and Trends. Biosensors.

[83] Zhang L., Wang X., Chen C., Wang R., Qiao X., Waterhouse G.I.N., Xu Z. (2023). A surface-enhanced Raman scattering sensor for the detection of benzo[a]pyrene in foods based on a gold nanostars@reduced graphene oxide substrate. Food Chem..

[84] Chen J., Lin H., Li S., Zhao J., Ahmed I., Zhi L., Li Z. (2021). Development of a Sandwich Enzyme-linked Immunosorbent Assay (ELISA) for the Detection of Egg Residues in Processed Food Products. Food Anal. Methods.

[85] Xi J., Shi Q., Lu Q. (2016). Development of an Indirect Competitive ELISA Kit for the Rapid Detection of Benzopyrene Residues. Food Anal. Methods.

[86] Jeeno P., Yadoung S., Thongkham M., Yana P., Jaitham U., Ounjaijean S., Xu Z.-L., Sringarm K., Hongsibsong S. (2024). In-House Immunoglobulin Y-Based Immunoassay for Detecting Benzo[a]pyrene in Grilled Pork Samples. Biosensors.

[87] Li W., Xu Z., He Q., Pan J., Zhang Y., El-Sheikh E.-S.A., Hammock B.D., Li D. (2025). Nanobody-Based Immunoassays for the Detection of Food Hazards—A Review. Biosensors.

[88] Xiao X., Hu S., Lai X., Peng J., Lai W. (2021). Developmental trend of immunoassays for monitoring hazards in food samples: A review. Trends Food Sci. Technol..

[89] Lee B., Park B., Kim D., Jung C., Park J.H., Park J.-H., Lee Y.E., Shin M.G., Kim M.-G., Yu N.E. (2025). Lateral flow immunoassay using plasmonic scattering. Nat. Commun..

[90] Yuan Y., Tang X., Zhang L., Zhang Q., Ma F., Li P. (2025). Multi-mode probe based on dual colorimetric and photothermal later flow immunoassay for the ultrasensitive determination of benzo[a]pyrene in vegetable oils. Food Chem..

[91] Soares W.M., Botelho B.G. (2025). Determination of benzo[a]pyrene in barley malt using constant wavelength synchronous fluorescence. J. Food Compos. Anal..

[92] Orfanakis E., Koumentaki A., Zoumi A., Philippidis A., Samartzis P.C., Velegrakis M. (2023). Rapid Detection of Benzo[a]pyrene in Extra Virgin Olive Oil Using Fluorescence Spectroscopy. Molecules.

[93] Liu W., Sun S., Xia Y., Zhao P., Liu C., Zheng L. (2022). Rapid Determination of Benzo(a)pyrene Concentration in Soybean Oil by Terahertz Transmission Spectroscopy with Chemometrics. J. Infrared Millim. Terahertz Waves.

[94] Yang J., Bai X., Wei M., Jiang H., Xu L. (2025). Terahertz Spectroscopy for Food Quality Assessment: A Comprehensive Review. Foods.

[95] Wang J.-p., Wang Y., Guo X., Wang P., Zhao T., Wang J. (2018). Matrix assisted laser desorption/ionization time-of-flight mass spectrometric determination of benzo[a]pyrene using a MIL-101(Fe) matrix. Microchim. Acta.

[96] Zhao X., Guo C., Huang Y., Huang L., Ma G., Liu Y., He Q., Wang H., Chen K., Pan Y. (2019). Combination Strategy of Reactive and Catalytic Matrices for Qualitative and Quantitative Profiling of N-Glycans in MALDI-MS. Anal. Chem..

[97] Linscheid M.W. (2019). Molecules and elements for quantitative bioanalysis: The allure of using electrospray, MALDI, and ICP mass spectrometry side-by-side. Mass Spectrom. Rev..

[98] Hu W., Zhang X., Shen Y., Meng X., Wu Y., Tong P., Li X., Chen H., Gao J. (2024). Quantifying allergenic proteins using antibody-based methods or liquid chromatography–mass spectrometry/mass spectrometry: A review about the influence of food matrix, extraction, and sample preparation. Compr. Rev. Food Sci. Food Saf..

[99] (2017). Animal and vegetable fats and oils—Determination of benzo[a]pyrene—Reverse-phase high performance liquid chromatography method.

[100] Adade S.Y.-S.S., Lin H., Nunekpeku X., Johnson N.A.N., Agyekum A.A., Zhao S., Teye E., Qianqian S., Kwadzokpui B.A., Ekumah J.-N. (2025). Flexible paper-based AuNP sensor for rapid detection of diabenz (a,h)anthracene (DbA) and benzo(b)fluoranthene (BbF) in mussels coupled with deep learning algorithms. Food Control.

[101] Mohan B., Priyanka, Singh G., Chauhan A., Pombeiro A.J.L., Ren P. (2023). Metal-organic frameworks (MOFs) based luminescent and electrochemical sensors for food contaminant detection. J. Hazard. Mater..

[102] Tomac I., Adam V., Labuda J. (2024). Advanced chemically modified electrodes and platforms in food analysis and monitoring. Food Chem..

[103] Wang Q., Chang K., Yang Q., Wu W. (2024). Semiconductor-based surface-enhanced Raman scattering sensing platforms: State of the art, applications and prospects in food safety. Trends Food Sci. Technol..

[104] Jayaprakash V., You J.B., Kanike C., Liu J., McCallum C., Zhang X. (2023). Determination of Trace Organic Contaminant Concentration via Machine Classification of Surface-Enhanced Raman Spectra. Environ. Sci. Technol..

[105] Sun X., Zhang X., Wang L., Li Y., Muir D.C.G., Zeng E.Y. (2022). Towards a better understanding of deep convolutional neural network processes for recognizing organic chemicals of environmental concern. J. Hazard. Mater..

[106] Xu M.-L., Gao Y., Han X.-X., Zhao B. (2022). Innovative Application of SERS in Food Quality and Safety: A Brief Review of Recent Trends. Foods.

